# Rapid elimination of cervical cancer while maintaining the harms and benefits ratio of cervical cancer screening: a modelling study

**DOI:** 10.1186/s12916-022-02631-7

**Published:** 2022-11-09

**Authors:** Erik E. L. Jansen, Inge M. C. M. de Kok, Sylvia Kaljouw, Erhan Demirel, Harry J. de Koning, Jan. A. C. Hontelez

**Affiliations:** 1 Department of Public Health, Erasmus MC, University Medical Center Rotterdam, Rotterdam, The Netherlands; 2 Heidelberg Institute of Global Health, Heidelberg University, Heidelberg, Germany

**Keywords:** Cervical cancer, Vaccination, Screening, Elimination, Harms-benefits ratio

## Abstract

**Background:**

Human papillomavirus (HPV) vaccination and intensifying screening expedite cervical cancer (CC) elimination, yet also deteriorate the balance between harms and benefits of screening. We aimed to find screening strategies that eliminate CC rapidly but maintain an acceptable harms-benefits ratio of screening.

**Methods:**

Two microsimulation models (STDSIM and MISCAN) were applied to simulate HPV transmission and CC screening for the Dutch female population between 2022 and 2100. We estimated the CC elimination year and harms-benefits ratios of screening for 228 unique scenarios varying in vaccination (coverage and vaccine type) and screening (coverage and number of lifetime invitations in vaccinated cohorts). The acceptable harms-benefits ratio was defined as the number of women needed to refer (NNR) to prevent one CC death under the current programme for unvaccinated cohorts (82.17).

**Results:**

Under current vaccination conditions (bivalent vaccine, 55% coverage in girls, 27.5% coverage in boys), maintaining current screening conditions is projected to eliminate CC by 2042, but increases the present NNR with 41%. Reducing the number of lifetime screens from presently five to three and increasing screening coverage (61% to 70%) would prevent an increase in harms and only delay elimination by 1 year. Scaling vaccination coverage to 90% in boys and girls with the nonavalent vaccine is estimated to eliminate CC by 2040 under current screening conditions, but exceeds the acceptable NNR with 23%. Here, changing from five to two lifetime screens would keep the NNR acceptable without delaying CC elimination.

**Conclusions:**

De-intensifying CC screening in vaccinated cohorts leads to little or no delay in CC elimination while it substantially reduces the harms of screening. Therefore, de-intensifying CC screening in vaccinated cohorts should be considered to ensure acceptable harms-benefits ratios on the road to CC elimination.

**Supplementary Information:**

The online version contains supplementary material available at 10.1186/s12916-022-02631-7.

## Background

In May 2018, the World Health Organization (WHO) Director-General issued a call for action to achieve global elimination of cervical cancer (CC) as a public health problem [1]. To reach the goal of a CC incidence rate under 4 per 100,000 women, the WHO defined targets of 90% coverage of high-risk human papillomavirus (HPV) vaccination, 70% coverage of two lifetime cervical screens, and 90% treatment of detected lesions by 2030 [1]. This global call to action has sparked interest for countries to reach these targets as fast as possible, and for several high-income countries, elimination is projected to be reached between 2028 and 2046 depending on screening coverage, test sensitivity, population structure, and vaccination coverage [2, 3].

Intensifying screening strategies has been identified as the most potent strategy to expedite elimination, whereas the effect of increasing vaccination coverage on the year of CC elimination has had limited effect in previous studies [3].

However, although cervical screening might prevent cancers or detect cancers at an earlier stage, it inevitably also brings harms to individual women who do not have CC, such as overdiagnosis, unnecessary referrals, and unnecessary treatments of lesions that would have never progressed to cancer [4, 5]. These harms can create substantial morbidity in otherwise healthy women, for example through anxiety of the result of the screening, colposcopy or treatment or through increased risk of pre-term birth after treatment [6, 7].

Striving towards the WHO goal of CC elimination through more intensified screening may affect harms-benefits ratios, as the population is exposed to more screening rounds. This is especially true for women in vaccinated cohorts, where expected benefits of screening will decline as women are protected against CC either directly through vaccination [8, 9], or indirectly through herd immunity [10, 11], yet the expected harms will largely remain unchanged. Although screening guidelines have been changed for young women [12], no recommendations are published for when these vaccinated cohorts reach older ages. Furthermore, many high-income countries are moving towards primary HPV screening [12, 13]. Although HPV screening has shown to be more effective and cost-effective than cytology screening, it also leads to a higher number of unnecessary referrals than cytology and thus will increase the harms as well [14].

To our knowledge, no modelling studies have addressed the delicate balance of achieving CC elimination early without deteriorating the harms-benefits ratio of screening within the context of different vaccination coverage and screening strategies. Therefore, our aim was to estimate the timing of CC elimination and the expected delay in reaching elimination by maintaining current harms-benefits ratios of screening, using the situation in the Netherlands as an example. The Netherlands is a high-income country, that introduced HPV vaccination in 2009 with a bivalent vaccine and achieved an average coverage of about 55% of 12-year-old girls between 2015 and 2019 [15–17]. Furthermore, the Dutch population-based cervical screening programme replaced cytology with the HPV test as the primary screening test in January 2017 [13]. We used a hybrid model of two well-established microsimulation models for HPV transmission (STDSIM) [18] and CC screening (MISCAN) [14] quantified to the Dutch situation and simulated the timing of CC elimination and the harms-benefits ratios of 228 unique scenarios of vaccination and screening.

## Methods

### Models

STDSIM is an individual-based model that simulates a dynamic sexual network through which sexually transmitted infections, such as HPV, can be transmitted. The model has been quantified to represent the Dutch heterosexual network [18] and has been used in the past to study the impact of gender-neutral and adult HPV vaccination on HPV 16 and 18 and cervical cancer in the Netherlands [19, 20]. For the purpose of this study, we re-calibrated HPV transmission probabilities, durations, and natural immunity development for HPV16 and HPV18, and a combined type representing the other five high-risk nonavalent types (HPVh5: HPV31, 33, 45, 52, and 58) to reproduce observed HPV type-specific prevalence as identified in the Dutch national screening programme. More details on the calibration procedure can be found in Additional file 1 (pp. 2‒3, Fig. S1 and Tables S1 and S2) [1, 13–18, 20–30]. The model assumes all vaccines to have a 95% lifelong efficacy against either only HPV16 and HPV18 (bivalent vaccine) or HPV16, HPV18, and HPVh5 (nonavalent vaccine). In a sensitivity analysis, we assumed efficacy to wane. Duration of protection for each individual is drawn from a Weibull distribution with a scale of 30 years and a shape of 5.

The effects of vaccination in the form of relative incidence reductions by type, birth cohort, age and vaccination status as estimated by STDSIM were applied as relative reductions in HPV infections in the second model, MISCAN-Cervix (Microsimulation Screening Analyses-Cervix). MISCAN-Cervix simulates the natural history of cervical cancer and the effects of cervical screening in a hypothetical population of women which are at risk for acquiring a high-risk HPV infection. This infection may clear or progress sequentially to a cervical intraepithelial neoplasia (CIN) grade 1, CIN2, CIN3 and cervical cancer or regress at any time until it has become cervical cancer. MISCAN-Cervix distinguishes between four categories of HPV types: HPV16, HPV18, other HPV types covered by the nonavalent vaccine (i.e. HPV-31/33/45/52/58), and the remaining high-risk HPV types (i.e. HPV-35/39/51/56/59/66/68) [14]. Progression probabilities of the disease depend on the HPV category, the current lesion grade and the age of the women [14]. Test characteristics of both the HPV test and cytology are dependent on the lesion grade as described previously [14]. The model simulates a population of women representative for the Dutch population in terms of birth rates, life expectancy and cervical cancer epidemiology. The simulations ran from 1912 to 2100, and cover a total of 100 million individual women. Life expectancy is assumed to remain constant over time with a median of 86.8 years [31]. A full model description including the calibration process to the Dutch female population was published previously [14].

### Simulated scenarios for vaccination and screening

For the base case scenario, we assumed that the vaccination coverage for girls will remain constant at 55% in the future, which is equal to the most recent 5-year average (Additional file 1 p. 4, Table S3) [15–17]. For all scenarios in our analysis, we introduced gender-neutral vaccination from 2021 onwards. Based on observations from other European countries [27], we assumed coverage for boys to be 50% of that of girls (Table 1). For screening, we simulated the Dutch programme as described in Additional file 1 (pp. 5‒6, Figs. S2 and S3, Table S4), including the switch from cytology to primary HPV testing in 2017 [13], with an adherence to primary testing of 61% (Table 1) which was not correlated with vaccination status, and imperfect adherence to follow-up [14]. We assumed that 10% of the population never attends screening [28]. All screening tests to be performed under a certain coverage assumption are then divided over the remaining 90% of the population (e.g. 61% coverage on the population level is achieved by letting 90% of the population attend 61%/90% = 67.78% of the received invitations). In a sensitivity analysis, we also re-ran all our scenarios assuming complete random non-attendance (e.g. 61% adherence on the population level is achieved by letting 100% of the population attend 61% of the received invitations). Women are invited for five lifetime HPV screens, at ages 30, 35, 40, 50 and 60, but may receive additional invitations at ages 45, 55, and 65 depending on attendance and screening results in the previous round (Table 1). To facilitate comparison with scenarios in which screening is scaled up, we assumed that screening participation is equal across all ages and all participating women participate by having a smear at their GP and adhere to follow-up advice as in the current situation.

**Table 1:** Simulated scenarios main analyses. In all vaccination scenarios, girls are invited for vaccination with the bivalent vaccine since 2009 and gender-neutral vaccination is implemented from 2021. Vaccination coverage in boys is 50% of those in girls, unless indicated otherwise

	Base case	Alternative scenarios, changes apply from 1 January 2022 onwards
Vaccination coverage in girls^a^	55%	60%, 70%, 80%, 90%, 90%^b^
Vaccine type	Bivalent	Nonavalent
Screening coverage^c^	61%	70% Stop screening for all women
Number of lifetime screens for either vaccinated women or vaccinated cohorts (screening ages)	5 (at 30, 35, 40, 45^d^, 50, 55^d^, 60, and 65^d^ years) [13]	0 (No screening) 1 (at 40 years) [29] 2 (at 35 and 45 years) [1] 3 (at 35, 47 and 59 years) [29]

^a^ Vaccination coverage of boys is assumed to be 50% of that in girls

^b^ In this scenario, coverage of boys is also assumed to be 90%

^c^ Distributed over 90% of the population. The remaining 10% of the population is assumed never to attend screening. This assumption is removed in a sensitivity analysis

^d^ screening age only applies if a woman was HPV-positive in the previous round (45, 55 and 65) or did not attend the previous round (45 and 55) as in the current Dutch programme [13]

We simulated a total of 228 scenarios, consisting of the base-case scenario and 227 scenarios in which changes are applied from 1 January 2022 onwards (Table 1). These scenarios include all combinations of alternative vaccination coverage, vaccination type, screening coverage and number of lifetime screens for either vaccinated women or entire vaccinated cohorts. Vaccination coverage in girls ranges from 55% to the WHO goal of 90%, with coverage in boys to be 50% of that in girls. Furthermore, we added an extreme scenario in which the coverage in boys and girls is increased to 90% [1, 27]. Vaccination type was either the bivalent or the nonavalent vaccine and screening coverage was either the current 61% or the WHO goal of 70% [1]. The number of lifetime screens for either vaccinated women or entire vaccinated cohorts was either zero, one, two, three or five.

For two lifetime screens, we invited women at ages 35 and 45, as proposed in the WHO goal [1]. For one and three lifetime screens, we invited women at age 40 and at ages 35, 47 and 59, respectively, as was found optimal in a previous study [29]. Furthermore, for each vaccination scenario, a scenario was added in which screening is stopped for all women. More details on the adaptations made in the different scenarios can be found in Additional file 1 pp. 4‒6.

We realise that other modelling studies focussing on elimination examined timing to elimination with up to 90% screening coverage [3, 32, 33]. Because we deem this screening coverage unrealistic for the Netherlands in the current situation, we decided not to include this in the main analyses, but to include outcomes with 90% screening coverage in Additional file 1, Figs. S4–S39 to enhance comparability with previous studies.

### Main outcome measures

For each scenario, we defined the year of CC elimination as the first calendar year where the CC incidence, age-standardised to the WHO standard population [30], is below the 4 per 100,000 threshold and stays there for at least 5 years. Furthermore, for each strategy, we assess the steady state incidence rates, defined as the average standardised incidence rate over the last 5 years of the simulation and the number of CC deaths per 100,000 life years over the period 2022–2100.

To assess the harms-benefits ratio of screening over the period 2022–2100, we divided the total number of referrals to a gynaecologist in that period by the total number of prevented CC deaths compared to a no-screening counterfactual with the same vaccination (number needed to refer, NNR). We used CC deaths instead of CC incidences as the denominator because of the potential effects of less frequent screening on stage shifts in detected cancers (i.e. with less frequent screening, detected cancers could on average be of more advanced stages, thereby increasing CC mortality). The harms-benefits ratio was defined as acceptable if lower than that of the current Dutch screening programme in unvaccinated cohorts. For each vaccination scenario, we selected the optimal screening strategy, which we defined as the screening strategy that reaches elimination first within the strategies with an acceptable harms-benefits ratio. If multiple strategies fulfilled these criteria, we selected the strategy with the largest mortality reduction compared to the current situation. Additionally, we determined the number needed to screen (NNS) per prevented cancer death, because the NNS can be seen as a proxy for the efficiency of the programme, as screening tests make up the largest part of the total costs of a programme [14].

We have completed the HPV-FRAME checklist of recommended reporting standards for HPV modelling studies as published by Canfell et al. [34] and included this checklist in Additional file 2 [14, 18, 34, 35].

## Results

**Fig. 1:**
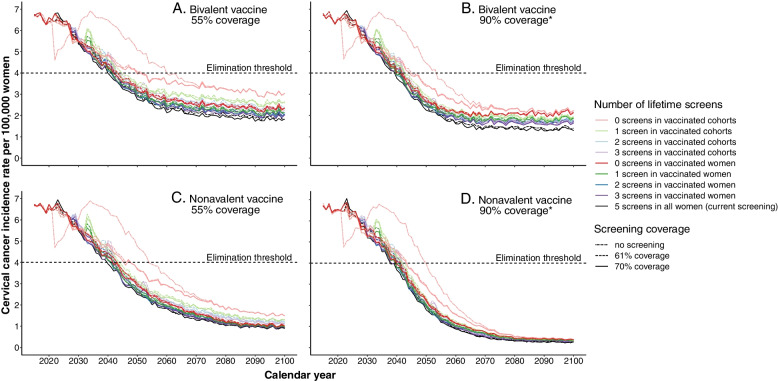
Predicted cervical cancer incidence rates in the Netherlands over the period 2022–2100. The incidence rates of the different screening scenarios are presented separately for four vaccination scenarios: **A** 55% vaccination coverage in girls and 27.5% in boys with a bivalent vaccine; **B** 90% coverage in boys and girls with a bivalent vaccine; **C** 55% vaccination coverage in girls and 27.5% in boys with a nonavalent vaccine; **D** 90% coverage in boys and girls with a nonavalent vaccine. Results are standardised to the WHO standard population [30]. * Coverage in boys and girls are both assumed to be 90%, whereas in the other scenarios the coverage in boys is assumed to be 50% of that in girls

Figure 1 shows the CC incidence rates over time for the four most extreme vaccination scenarios. All of the 228 simulated scenarios reach the CC elimination threshold before the year 2100. In the scenario where both screening and vaccination remain as it is, CC elimination will be reached in the year 2042. In scenarios that maintain the current vaccination baseline (bivalent vaccine with 55% coverage; Fig. 1A), the fastest elimination (in 2040) is achieved when maintaining current screening strategies of 5 lifetime screens, at 70% coverage. Reducing screening intensity delays the year of elimination, yet most strategies will have reached elimination by 2045. Only when screening for vaccinated cohorts is stopped entirely, elimination is estimated to be delayed to 2052 (orange lines in Fig. 1A). Fastest elimination is still reached in 2040 when vaccination coverage is increased to 90% in boys and girls (Fig. 1B), yet now most scenarios will have reached elimination by 2043, and ending screening for vaccinated cohorts entirely will delay elimination until 2048 if screening coverage could not be increased in the unvaccinated cohorts. The steady-state incidence rates would be 24% to 25% lower than when the current vaccination coverage would be maintained.

Switching to a nonavalent vaccine while maintaining the current vaccination coverage (Fig. 1C) will only substantially expedite elimination for the scenario in which screening would be stopped in vaccinated cohorts (to 2048), yet steady state incidence rates are reduced by 49% compared to the same vaccination coverage with the bivalent vaccine. Combining the switch to a nonavalent vaccine with increasing vaccination coverage to 90% in boys and girls (Fig. 1D) could reach elimination fastest, i.e. in 2038, when maintaining five lifetime screens and a coverage increase to 70%. Stopping screening in vaccinated cohorts would still reach elimination in 2044, and steady-state incidence rates would decrease by 87% compared to the base case vaccination scenarios.

**Fig. 2:**
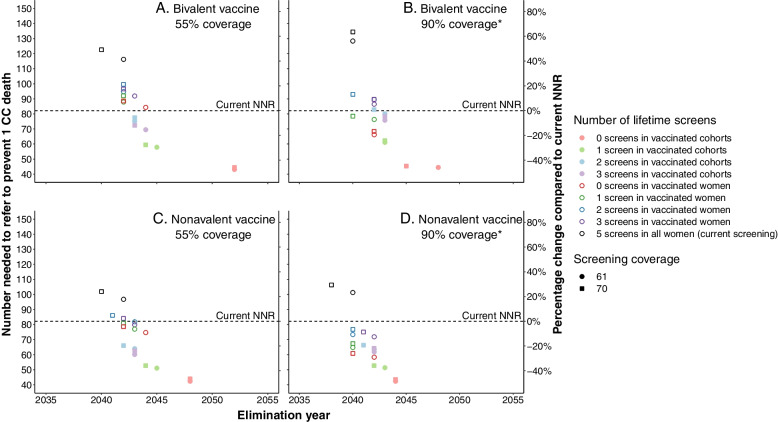
NNR to prevent one cervical cancer death and the CC elimination year for each screening strategy and the percentage change to the NNR of the current strategy in unvaccinated cohorts. The results are presented for the Dutch cervical cancer screening programme over the period 2022–2100 for four vaccination scenarios: **A** 55% vaccination coverage in girls and 27.5% in boys with a bivalent vaccine; **B** 90% coverage in boys and girls with a bivalent vaccine; **C** 55% vaccination coverage in girls and 27.5% in boys with a nonavalent vaccine; **D** 90% coverage in boys and girls with a nonavalent vaccine. The horizontal dotted line represents the current NNR to prevent one CC death for unvaccinated cohorts. NNR, number needed to refer to prevent 1 CC death; CC, cervical cancer. * Coverage in boys and girls are both assumed to be 90%, whereas in the other scenarios the coverage in boys is assumed to be 50% of that in girls

Figure 2 shows the correlation between the NNR to prevent one CC death and the year of reaching elimination for each screening strategy for four vaccination scenarios. Figure 2A and B show the results for the bivalent vaccine with 55% and 90% coverage among girls respectively (27.5% and 90% coverage respectively among boys). Figure 2C and D show the results for the nonavalent vaccine with the same respective vaccination coverages. The dotted black lines in Fig. 2 represent the current NNR for unvaccinated cohorts in the Netherlands.

Overall, the NNR is higher for scenarios with an early elimination year than for those with a later elimination year. Under the current vaccination policy (Fig. 2A), maintaining the current screening strategy would lead to a 41% higher NNR compared to the current programme in unvaccinated women. Continuing the current five lifetime screens and increasing screening coverage to 70% reaches elimination fastest, but leads to a 49% increase in NNR. Earliest elimination without increasing the NNR could be reached in 2043. Increasing vaccination coverage to 90% in girls and boys (Fig. 2B) leads to a decrease in NNR for most screening strategies compared to the same strategies with 55% vaccination coverage among girls and 27.5% coverage among boys. Here, the fastest elimination would not increase the NNR, provided that vaccinated women receive only one lifetime screen and screening coverage reaches 70% (open green square in Fig. 2B).

Switching to the nonavalent vaccine at 55% coverage (Fig. 2C) would also decrease the NNR for most screening strategies compared to the same strategies with the bivalent vaccine, resulting in the fastest elimination with an NNR below current levels in 2042. Switching to a nonavalent vaccine at 90% coverage in girls and boys would reduce the NNR to below the current NNR for all screening strategies except for those with five lifetime screens (+ 23% NNR at 61% screening coverage. The fastest elimination without exceeding the current NNR is can be reached in 2040.

The incidence and NNR graphs of the remaining vaccination scenarios and the 90% screening attendance scenarios are presented in Additional file 1 Figs. S4‒S7. Reaching 90% screening attendance will eliminate cervical cancer earlier, but increases the NNR in all vaccination scenarios. Therefore, screening needs to be further de-intensified to maintain acceptable levels of NNR.

**Fig. 3:**
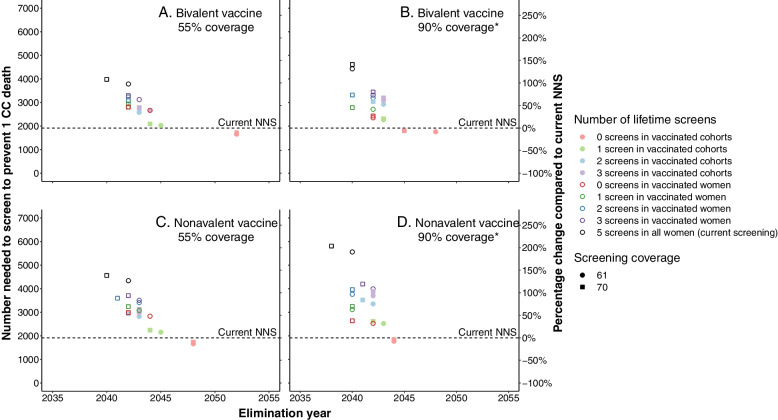
NNS to prevent one cervical cancer death and the CC elimination year for each screening strategy and the percentage change to the NNS of the current strategy in unvaccinated cohorts. The results are presented for the Dutch cervical cancer screening programme over the period 2022–2100 for four vaccination scenarios: **A** 55% vaccination coverage in girls with a bivalent vaccine; **B** 90% coverage in boys and girls with a bivalent vaccine; **C** 55% vaccination coverage in girls with a nonavalent vaccine; **D** 90% coverage in boys and girls with a nonavalent vaccine. The horizontal dotted line represents the current NNR to prevent one CC death for unvaccinated cohorts. CC, cervical cancer; NNS, number needed to screen to prevent 1 CC death. * Coverage in boys and girls are both assumed to be 90%, whereas in the other scenarios the coverage in boys is assumed to be 50% of that in girls

Figure 3 shows that, regardless of vaccination strategy, the NNS to prevent one CC death exceeds the current NNS in the Netherlands (dotted black line in Fig. 3A‒D) for all strategies except when stopping screening for vaccinated cohorts (orange markers in Fig. 3A‒D). In these scenarios, elimination is projected to be delayed four to ten years compared to the current screening strategy, depending on the vaccination scenario. For all other screening strategies, the NNSs are increased up to 204%, compared to the NNS in unvaccinated cohorts.

The NNS graphs of the remaining vaccination scenarios are presented in Additional file 1 Figs. S28‒S39.

Table 2 presents the optimal screening strategy that reaches elimination first under acceptable NNRs for each vaccination scenario, the delay in reaching elimination by maintaining NNRs, and the resulting CC deaths prevented compared to the current screening programme in unvaccinated women. In each vaccination scenario, elimination can be reached in 2043 or earlier without exceeding the current NNR, and elimination is only delayed between 0 and 3 years compared to the fastest possible elimination for each vaccination scenario. De-intensified screening would result in slightly more CC deaths with the bivalent vaccine under sub-optimal coverage levels (3% to 5% increase). However, switching to a nonavalent vaccine or increasing vaccination coverage to 90% would still result in a reduction in CC deaths (ranging from 1 to 22%) even when de-intensifying screening to maintain current NNRs. Although all optimal scenarios contain a lower screening frequency than the current five lifetime screens, eleven out of twelve optimal scenarios contain the increased screening coverage of 70%.

**Table 2:** Optimal screening strategies that reach elimination first without exceeding the current NNR, by vaccination scenario

Vaccination scenario		Screening strategy for vaccinated cohorts^b^		Elimination year (years of delay^c^)	Cervical cancer deaths per 100,000 person years over 2022–2100	
Coverage^a^	Vaccine type	Number of lifetime screens	Coverage (%)		Optimal strategy	Difference with base case scenario^d^
90%^e^	9 V	2^f^	70	2040 (2)	1.21	− 0.34 (− 22%)
90%	9 V	2^f^	70	2040 (1)	1.24	− 0.32 (− 20%)
80%	9 V	2^f^	70	2040 (0)	1.27	− 0.29 (− 19%)
70%	9 V	2^f^	70	2041 (1)	1.31	− 0.25 (− 16%)
60%	9 V	1^f^	70	2041 (1)	1.38	− 0.17 (− 11%)
55%	9 V	1^f^	70	2042 (2)	1.44	− 0.11 (− 7%)
90%^e^	2 V	1^f^	70	2040 (0)	1.53	− 0.03 (− 2%)
90%	2 V	1^f^	70	2042 (2)	1.55	− 0.01 (− 1%)
80%	2 V	1^f^	61	2042 (2)	1.60	+ 0.04 (3%)
70%	2 V	3	70	2043 (3)	1.54	− 0.02 (− 1%)
60%	2 V	3	70	2043 (3)	1.60	+ 0.04 (3%)
55%	2 V	3	70	2043 (3)	1.64	+ 0.08 (5%)

^a^ Vaccination coverage in girls. Coverage in boys is assumed to be 50% of that in girls unless indicated otherwise

^b^ Unvaccinated cohorts are screened according to the current guidelines

^c^ Years of delay compared with the most intense screening strategy (five lifetime screens for all women and 70% screening coverage)

^d^ In the base case scenario vaccination coverage is 55% with the bivalent vaccine. Women receive 5 lifetime screening invitations and screening coverage is 61%

^e^ 90% coverage in both boys and girls

^f^ Women in vaccinated cohorts that did not receive vaccination are screened to the current guidelines

9 V, nonavalent vaccine; 2 V, bivalent vaccine; NNR, number needed to refer to prevent 1 cervical cancer death

In Additional file 1, we present all optimal strategies for the sensitivity analyses where we either assumed complete random non-attendance to screening instead of 10% consistent non-attendance (Table S8) or waning vaccination (Table S9). In both sensitivity analyses, screening needs to be de-intensified to maintain current NNRs and the maximum elimination delay by de-intensified screening remains 3 years. Assuming random non-attendance expedites elimination by 3 to 9 years compared to the base case scenarios, whereas assuming waning vaccination delays elimination with 1 year in some of the scenarios.

## Discussion

We have shown that elimination of CC in the Netherlands can be reached in 2042 under status quo conditions, but could be expedited to the year 2038 if 90% vaccination coverage with a nonavalent vaccine in boys and girls can be achieved, screening coverage reaches 70% and both vaccinated and unvaccinated women receive five lifetime HPV screening invitations. However, the latter scenario would result in a deterioration of the harms-benefits ratio of screening by 29% compared to the current situation. Our model predictions show that maintaining current harms-benefits ratios by de-intensifying screening in vaccinated cohorts would only delay elimination by up to 3 years, depending on the vaccination scenario. While de-intensifying screening frequency would slightly increase projected CC deaths under continued use of the bivalent vaccine, switching to nonavalent vaccination strategies or increasing vaccination to 90% would offset this loss in effectiveness of the screening programme and is projected to result in additional deaths prevented even under de-intensified screening strategies.

Our results demonstrate that, while reaching early elimination for CC is a noble endeavour, we should question whether it should be a core target for programme development for CC control policies. Our results show that the relative modest gains of a few years earlier elimination compared to de-intensified screening programmes could come at a severe cost of deteriorating harms-benefit ratios. This deterioration means an increase in referrals per death averted, which will in turn lead to more overdiagnosis and overtreatment. These conclusions were robust to alternative assumptions on screening behaviour and vaccine efficacy. What’s more, our model predictions also show that CC screening will likely become less efficient compared to the current situation as the NNS increases substantially. Because the screening tests make up the largest part of the total costs of a programme the cost-effectiveness will likely deteriorate as well [14]. The costs of these screens might be spent more efficiently in other areas of healthcare. Therefore, choosing elimination strategies is best done from a sub-set of de-intensified screening strategies that can roughly maintain or improve acceptable efficiency and harms-benefit ratio benchmarks.

Although the numbers in our results are based on the example of the Netherlands, our conclusion that screening needs to be de-intensified is generalizable to most other high-income countries. The expected year of elimination might differ between countries because of differences in implementation of vaccination such as implementation year and catch-up strategies, and differences in background risk such as screening history, but the expected increase in NNR and NNS for the current strategies will remain because of the high efficacy of the HPV vaccines, which decreases the benefits of screening for vaccinated cohorts. Furthermore, stratifying screening intensity by vaccination status might not be feasible for all countries because the vaccination status might not be available to the screening organization. In these cases, the strategies that apply de-intensified screening to entire cohorts that are offered vaccination can still be considered because only the birth year of the woman is required to apply such strategy. Alternatively, the benefits of stratified screening could help motivate a change in screening programmes so that vaccination records can be linked in countries where this is currently not possible.

In low-income and lower-middle-income countries (LMICs) however, the current CC burden is often much higher and most LMICs have not yet implemented an HPV vaccination programme. Vaccination is essential to reach elimination, especially in LMICs, but screening will remain an important addition there because elimination will not be reached in 40% of LMICs with vaccination alone. Furthermore, screening can expedite elimination in LMICSs by up to 31 years [32].

To our knowledge, this is the first modelling study that aimed to find screening strategies that reach CC elimination fast while balancing the harms and benefits of screening. Previous modelling studies have estimated the impact of various strategies on the timing of elimination [2, 3, 32, 36–39], of which three also included the cost-effectiveness of those strategies [37–39], but none looked at how these elimination strategies affected the harms-benefits ratios of screening. In a comparative modelling study on CC elimination, Burger et al. applied two independently-developed models to project the year of CC elimination in the USA [3]. Under status quo conditions, one model predicted CC elimination to be reached in 2038 and the other model predicted 2046. Although the status quo conditions in the USA are different than those in the Netherlands, the projected times to CC elimination were very similar. Another modelling study predicted that elimination will happen in Australia in 2028 under status quo conditions, which is 14 years earlier than this study predicts for the Netherlands [2]. This difference is likely to be caused by the differences in vaccination policy and coverage, as in Australia HPV vaccination started in 2007 instead of 2009, included a catch-up until age 26 instead of 16, includes boys from 2013 onwards and uses a nonavalent vaccine since 2018. Furthermore, the authors assumed a 100% vaccine efficacy instead of the 95% efficacy that was assumed in this study.

Our study has four limitations. First, the disruption in screening practice due to the COVID-19 pandemic has not been taken into account in our simulations. However, it has been demonstrated that the interruptions in cervical screening have a very limited effect on the CC incidence [40]. Furthermore, the pandemic delayed the inclusion of boys in the vaccination programme to 2022, although this effect might be mitigated by future catch-up programmes. Therefore, no major differences in conclusions are expected due to the pandemic, especially because any effects of the disruptions will be diluted over time. A second limitation is that the NNR and NNS of the scenarios were compared to their current values in the Netherlands where the current number of lifetime screens is already low compared to many other countries and therefore the NNR and NNS are already relatively low. Third, we did not consider other programme adjustments than de-intensifying screening for vaccinated cohorts to improve the harms-benefits ratio. Kaljouw and colleagues demonstrated that unnecessary referrals can be reduced by using genotyping in the triage strategy or by extending the time to a repeat test [35]. Both methods could be implemented complementary to each other. Lastly, it is yet unknown if Dutch women would accept less frequent screening and if there will be a correlation between participation in vaccination and screening participation.

## Conclusions

CC elimination in the Netherlands will likely be achieved between 2038 and 2043. Because of a reduced risk for CC in vaccinated cohorts, the harms-benefits ratio of screening will deteriorate if the number of lifetime screenings is not decreased for women in vaccinated cohorts. Therefore, programmatic planning towards CC elimination in high-income countries should carefully consider the effects of screening policies in vaccinated cohorts, on increasing harms-benefits ratios and reducing efficiency. While only causing a few years of delay, comprehensively developed de-intensified screening strategies based on the local epidemiological context would provide an efficient and acceptable path to reaching CC elimination within the next decades.

## Supplementary Information

**Additional file 1:** **Table S1.** Duration model parameters and 1-year persistence for HPV16, HPV18, and HPVh5 as used in STDSIM. **Table S2.** Ranges of transmission probabilities and acquired immunity that resulted in acceptable model fits to prevalence data by type. **Table S3. **Simulated vaccination coverage by birth year and sex for the base case scenario. **Table S4.** Simulated scenarios main analyses. **Table S5.** List of figures that present the incident rates over time with their corresponding vaccination coverage. **Table S6.** List of vaccination coverages of all figures that present the NNR of each screening strategy by elimination year. **Table S7.** List of vaccination coverages of all figures that present the NNS of each screening strategy by elimination year. **Table S8.** Optimal screening strategies if random non-attendance is assumed Table S9. Optimal screening strategies if waning vaccination efficacy is assumed. **Figure S1.** Model predicted age specific HPV prevalence levels by type. **Figure S2.** Triage in the simulated cytology screening programme. **Figure S3.** Triage in the simulated HPV screening programme. **Figures S4-S15.** Predicted cervical cancer incidence rates in the Netherlands over the period 2020—2100 for the different screening scenarios. Vaccination coverages in separate figures. **Figures S16-S27.** Predicted NNR of cervical cancer screening in the Netherlands over the period 2022—2100 by the year in which elimination will be reached for that strategy. Vaccination coverages in separate figures. **Figures S28-S39.** Predicted NNS of cervical cancer screening in the Netherlands over the period 2022—2100 by the year in which elimination will be reached for that strategy. Vaccination coverages in separate figures.

**Additional file 2.** HPV-FRAME reporting checklist.

## Data Availability

The datasets generated and analysed during the current study are available from the corresponding author on reasonable request.

## Abbreviations

HPV: Human papillomavirus
CC: Cervical cancer
NNR: Number needed to refer
WHO: World Health Organization
CIN: Cervical intraepithelial neoplasia
NNS: Number needed to screen
9V: Nonavalent vaccine
2V: Bivalent vaccine
LIMCs: Lower-income and lower-middle-income countries
