# Supplementary material for: Rapid elimination of cervical cancer while maintaining the harms and benefits ratio of cervical cancer screening: a modelling study
Source: BMC Med. 2022 Nov 9;20:433. doi: 10.1186/s12916-022-02631-7 (PMC9645325; doi:10.1186/s12916-022-02631-7)
Supplement: Supplementary file 1 — Additional file 1: Table S1. Duration model parameters and 1-year persistence for HPV16, HPV18, and HPVh5 as used in STDSIM. Table S2. Ranges of transmission probabilities and acquired immunity that resulted in acceptable model fits to prevalence data by type. Table S3. Simulated vaccination coverage by birth year and sex for the base case scenario. Table S4. Simulated scenarios main analyses. Table S5. List of figures that present the incident rates over time with their corresponding vaccination coverage. Table S6. List of vaccination coverages of all figures that present the NNR of each screening strategy by elimination year. Table S7. List of vaccination coverages of all figures that present the NNS of each screening strategy by elimination year. Table S8. Optimal screening strategies if random non-attendance is assumed Table S9. Optimal screening strategies if waning vaccination efficacy is assumed. Figure S1. Model predicted age specific HPV prevalence levels by type. Figure S2. Triage in the simulated cytology screening programme. Figure S3. Triage in the simulated HPV screening programme. Figures S4-S15. Predicted cervical cancer incidence rates in the Netherlands over the period 2020—2100 for the different screening scenarios. Vaccination coverages in separate figures. Figures S16-S27. Predicted NNR of cervical cancer screening in the Netherlands over the period 2022—2100 by the year in which elimination will be reached for that strategy. Vaccination coverages in separate figures. Figures S28-S39. Predicted NNS of cervical cancer screening in the Netherlands over the period 2022—2100 by the year in which elimination will be reached for that strategy. Vaccination coverages in separate figures. [file 12916_2022_2631_MOESM1_ESM.docx]

Content

[Calibration procedure STDSIM 2](#_Toc116391179)

[Simulating the current Dutch vaccination programme and the alternative vaccination strategies 4](#_Toc116391180)

[Simulating the Dutch cervical cancer screening programme and the alternative screening strategies 5](#_Toc116391181)

[Incidence rates over time for all vaccination scenarios 7](#_Toc116391182)

[NNR by elimination year for all vaccination scenarios 14](#_Toc116391183)

[NNS by elimination year for all vaccination scenarios 21](#_Toc116391184)

[Assuming random non-attendance 28](#_Toc116391185)

[Assuming waning vaccination 29](#_Toc116391186)

# Calibration procedure STDSIM

We recalibrated the STDSIM model to reproduce observed age-specific human papillomavirus (HPV) prevalence levels from the first round of the Dutch national screening programme that used a primary HPV test.(13, 21) While we had previously calibrated the HPV16 and HPV18 transmission probabilities, durations, and natural immunity responses for the Netherlands,(18, 20) they were calibrated against data from the POBASCAM trial,(22) which was later found to have a prevalence level that was not representative for the general Dutch situation. The first round of screening using a primary HPV test provided us with data from nearly 500 000 Dutch women nationwide for HPV16, HPV18, and other high risk (OHR) HPV types.(21) Based on the observations by Coupé et al,(23) we assumed that 53% of the OHR category consisted of types 31, 33, 45, 52, and 58, i.e. the five other high-risk types covered by the nonavalent vaccine (HPVh5), and captured in the HPVh5 type in STDSIM. For the age group 15 to 29, we used data from Mollers *et al*.(24)

We calibrated our model to reproduce the observed prevalence levels by sampling values for transmission probabilities, durations, and natural immunity for each type. Calibration is done by developing unique parameter sets through repeated Monte-Carlo sampling of parameter values from pre-defined distributions, and testing whether model predictions of HPV prevalence are acceptably close to the data. For each type, we repeat this procedure until we found 80 parameter combinations that produced acceptable HPV prevalence predictions. Model predictions were deemed acceptable when HPV prevalence in the 15-29 year age group fell within +/- 40% of the data point estimate, and at least four out of the five prevalence estimates for the older age groups fell within +/- 20% of the data point estimates. The resulting ranges of uncertainty produced by these acceptability bounds are shown in Figure S1.


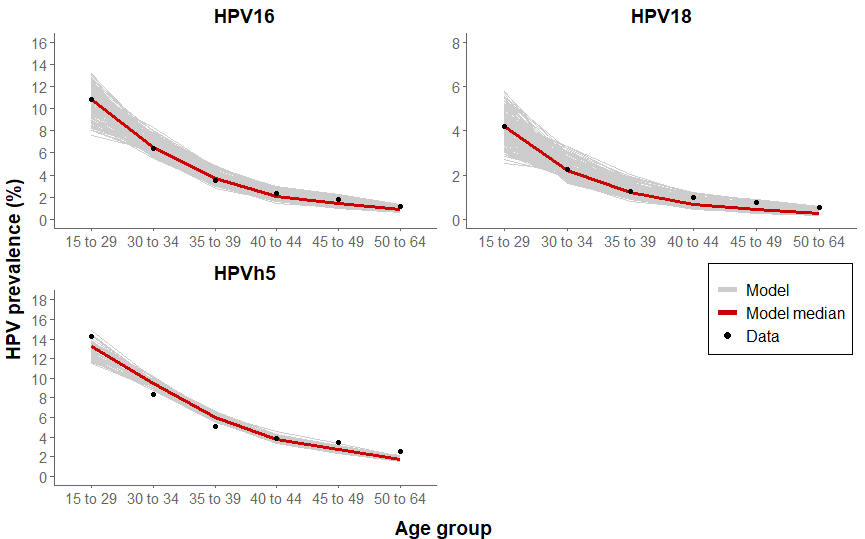


**Figure S1. Model predicted age specific HPV prevalence levels by type. Grey lines represent individual model parameter combinations, while the red line represents the median prediction of all individual parameter combinations. The black dots represent the observed prevalence in the Dutch primary HPV screening programme between 1 January 2017 and 9 March 2018.**

In the calibration exercise, transmission probabilities and level of natural immunity after infection were free parameters (i.e. values were sampled from the full range of possible values: 0.0 to 1.0), while the duration of infection is informed by studies from Ramanakumar *et al*.(25) In our model, natural immunity is partial,(26) and stacks proportionally with every subsequent reinfection. We incorporate no waning of natural immunity.

First, we developed a range of parameter values that result in simulated HPV durations within the confidence intervals of the study by Ramanakumar *et al*.(25) In STDSIM, each individual that is newly infected will be assigned a duration for that infection drawn from a user defined probability distribution. For HPV, infection durations are drawn from Weibull distributions, and we determined which combinations of shape and scale of the Weibull distribution would result in an mean duration, median duration, and 1-year persistence within the confidence intervals of the data from Ramanakumar *et al*.(25) The resulting ranges of Weibull parameters and durations of are given in Table S1.

**Table S1. Duration parameters and resulting median and mean duration, and 1-year persistence for HPV16, HPV18, and HPVh5 as used in STDSIM.**

|  | **Weibull Scale range** | **Weibull shape range** | **Median duration range (months)** | **Mean duration range (months)** | **1-year persistence range (proportion)** |
| --- | --- | --- | --- | --- | --- |
| HPV16 | 0.9; 2.2 | 14.1; 18.7 | 10.3; 16.7 | 13.0; 18.5 | 0.39; 0.61 |
| HPV18 | 0.7; 2.1 | 9.2; 12.2 | 5.8; 11.0 | 8.3; 13.0 | 0.26; 0.38 |
| HPVh5 | 0.7; 2.2 | 10.0; 12.7 | 6.3; 12.4 | 9.5; 14.7 | 0.29; 0.42 |

In the next step, we calibrated our model to reproduce observed HPV prevalence levels. For each of the Weibull scale and shape combinations for the durations of the HPV types, we repeatedly sampled random combinations of per-act transmission probabilities (ranging from 0.0 to 1.0) and level of acquired immunity after clearance (ranging from 0.0 to 1.0, where 0.0 is no immunity, and 1.0 is full immunity) until we found a combination that produced prevalence levels within the predefined acceptable ranges. Table S2 shows the resulting ranges in transmission probabilities and levels of acquired immunity after clearance; Figure S1 shows the resulting fit of the model for all unique combinations (grey lines) and the median prediction over all combinations (red line). The median prediction (red line) was used to produce the estimates for the current study.

**Table S2. Ranges of transmission probabilities and acquired immunity that resulted in acceptable model fits to prevalence data by type.**

|  | **Transmission probability range** | **Level of acquired immunity range** |
| --- | --- | --- |
| HPV16 | 0.035; 0.075 | 0.043; 0.351 |
| HPV18 | 0.040; 0.101 | 0.004; 0.679 |
| HPVh5 | 0.059; 0.089 | 0.011; 0.073 |

# Simulating the current Dutch vaccination programme and the alternative vaccination strategies

HPV vaccination was implemented in the Netherlands in 2009 with a bivalent vaccine. In STDSIM we simulated the observed coverage over the birth years up to 2005 as presented in Table S3.(15-17) For the succeeding birth years for which no data was available yet, we assumed that the coverage in girls would remain constant at the most recent 5-year average (birth years 2001-2005, 55,2% coverage). Vaccination in boys was introduced in 2021 for birth year 2009 and up. No observed data on coverage among boys was available yet. Based on observations from other European countries,(27) we assumed coverage for boys to be 50% of that of girls, so 27.6% (Table S3).

**Table S3. Simulated vaccination coverage by birth year and sex for the base case scenario. All vaccinations are with the bivalent vaccine.**

| **Birth year** | **Coverage in girls** | **Coverage in boys** |
| --- | --- | --- |
| ≤1992 | 0 | 0 |
| 1993 | 0.49 | 0 |
| 1994 | 0.525 | 0 |
| 1995 | 0.538 | 0 |
| 1996 | 0.542 | 0 |
| 1997 | 0.56 | 0 |
| 1998 | 0.581 | 0 |
| 1999 | 0.589 | 0 |
| 2000 | 0.61 | 0 |
| 2001 | 0.61 | 0 |
| 2002 | 0.534 | 0 |
| 2003 | 0.455 | 0 |
| 2004 | 0.455 | 0 |
| 2005 | 0.72 | 0 |
| 2006 | 0.552 | 0 |
| 2007 | 0.552 | 0 |
| 2008 | 0.552 | 0 |
| 2009 | 0.552 | 0.276 |
| 2010 | 0.552 | 0.276 |
| 2011 | 0.552 | 0.276 |
| 2012 | 0.552 | 0.276 |
| ≥2013 | 0.552 | 0.276 |

For the alternative vaccination scenarios, we increased the vaccination coverage in girls from birth year 2013 onwards to either 60%, 70%, 80% or 90% and adjusted the coverage in boys to 30%, 35%, 40% and 45% respectively so that it would remain 50% of that in girls. Furthermore, we added a vaccination scenario in which coverage in girls and boys would both be increased to 90% (Table S4). Lastly, we simulated scenarios in which a switch would be made to the nonavalent vaccine for girls in birth year 2013 and up for the current vaccination coverage and all increased vaccination coverages (Table S4).

# Simulating the Dutch cervical cancer screening programme and the alternative screening strategies

Cervical cancer screening in the Netherlands has switched from a primary cytology programme to a primary HPV test programme in January 2017. We therefore simulated the Dutch cytology programme for women that were eligible for screening between the calendar years 1990-2016. Women were invited for screening at ages 30, 35, 40, 45, 50, 55 and 60. After a positive screening test, women were referred according to the triage scheme in Figure S2.

**Figure S2. Triage in the simulated cytology screening programme (Source: Jansen et al. 2020).(14)**

t = Time in months since primary test; OSP = Organised screening programme; HPV = Human papillomavirus; NILM = Negative for intraepithelial lesion or malignancy; ASC-US = Atypical squamous cells of undetermined significance; LSIL = Low-grade squamous intraepithelial lesion; HSIL = High-grade squamous intraepithelial lesion.

From January 2017, we simulated a primary HPV programme in which women are invited for five lifetime screens at ages 30, 35, 40, 50 and 60. Women that do not attend at age 40 or 50 will receive an extra invitation at age 45 or 55 respectively and women who test positive on the primary HPV test at age 40, 50 or 60 will receive an extra invitation at age 45, 55 or 65 respectively. The triage scheme of the simulated primary HPV programme is presented in Figure S3. Although in the Dutch cervical screening programme it is also possible to participate with self-sampling, we assumed all participation in screening to be by a smear at the GP to facilitate comparison with the simulated alternative strategies.

Participation in screening until January 2022 and adherence to follow-up advice was modelled as described previously.(14) In our base case scenario, we assumed that participation would remain constant at the observed average of 61%.(14) We assumed that 10% of the population never attends screening.(28) All screening tests to be performed in the base case scenario or any other scenario are divided over the remaining 90% of the population (e.g. 61% coverage on the population level is achieved by letting 90% of the population attend 61% / 90% = 67.78% of the received invitations). To facilitate comparison with other screening scenarios we assumed this participation to be independent of age. Furthermore we assumed the participation rate to be independent of vaccination status.

**Figure S3. Triage in the simulated HPV screening programme.**

t = Time in months since primary test; OSP = Organised screening programme; HPV = Human papillomavirus; - = Negative test result; + = Positive test result (i.e. ≥ ASC-US for cytology); NILM = Negative for intraepithelial lesion or malignancy; ASC-US = Atypical squamous cells of undetermined significance.

In the alternative screening scenarios, the lifetime number of lifetime screening invitations was reduced and/or screening participation after an invitation was increased from January 2022 onwards (Table S4). Strategies with a reduced number of lifetime screening invitations (i.e. three, two, one or zero) were implemented for either vaccinated women only or entire cohorts that were offered the vaccine. For two lifetime screens we invited women at age 35 and 45, as proposed in the WHO goal.(1) For one and three lifetime screens we invited women at age 40 and at ages 35, 47 and 59, respectively, as was found optimal in a previous study.(29) Screening participation was increased to the WHO goal of 70%.(1) Furthermore, we added a scenario in which screening is stopped for all women to be able to calculate the benefits of screening.

**Table S4. Simulated scenarios main analyses. In all vaccination scenarios, girls are invited for vaccination with the bivalent vaccine since 2009 and gender-neutral vaccination is implemented from 2021. Vaccination coverage in boys is 50% of those in girls, unless indicated otherwise.**

|  | Base case | Alternative scenarios, changes apply from 1 January 2022 onwards |
| --- | --- | --- |
| Vaccination coverage in girls^a^ | 55% | 60%, 70%, 80%, 90%, 90%^b^ |
| Vaccine type | Bivalent | Nonavalent |
| Screening coverage^c^ | 61% | 70%  Stop screening for all women |
| Number of lifetime screens for either vaccinated women or vaccinated cohorts (screening ages) | 5 (at 30, 35, 40, 45^d^, 50, 55^d^, 60, and 65^d^ years)(13) | 0 (No screening)  1 (at 40 years)(30)  2 (at 35 and 45 years)(1)  3 (at 35, 47 and 59 years)(29) |

^a^ Vaccination coverage of boys is assumed to be 50% of that in girls.

^b^ In this scenario, coverage of boys is also assumed to be 90%.

^c^ Distributed over 90% of the population. The remaining 10% of the population is assumed never to attend screening. This assumption is removed in a sensitivity analysis.

^d^ screening ages only applies if a women was HPV-positive in the previous round (45, 55 and 65) or did not attend the previous round (45 and 55) as in the current Dutch programme.(13)

# Incidence rates over time for all vaccination scenarios

Figures S4-S15 below present the incidence rates over time in the Netherlands for the different screening scenarios. Each graph represents a different vaccination scenario for newly vaccinated girls (Table S5). It is assumed that boys will have half the vaccination coverage of that of girls unless indicated otherwise. The results of the screening scenarios where a more ambitious goal of 90% screening coverage would be reached are included in these graphs as well. Reaching this ambitious goal would bring elimination forward compared to a 70% coverage for most screening strategies. In the current vaccination scenario elimination will be reached fasted in 2038 with 5 lifetime screens. If vaccination would be scaled up to either a 90% coverage in boys and girls with the bivalent vaccine or at least 60% coverage with a nonavalent vaccine, elimination could be reached in 2037 with 90% coverage and 5 lifetime screens.

**Table S5. List of figures that present the incident rates over time with their corresponding vaccination coverage.**

| **Figure** | **Vaccine type** | **Coverage in girls after 1 January 2022** | **Coverage in boys after 1 January 2022** |
| --- | --- | --- | --- |
| S4 | Bivalent | 55.2% | 27.6% |
| S5 | Bivalent | 60% | 30% |
| S6 | Bivalent | 70% | 35% |
| S7 | Bivalent | 80% | 40% |
| S8 | Bivalent | 90% | 45% |
| S9 | Bivalent | 90% | 90% |
| S10 | Nonavalent | 55.2% | 27.6% |
| S11 | Nonavalent | 60% | 30% |
| S12 | Nonavalent | 70% | 35% |
| S13 | Nonavalent | 80% | 40% |
| S14 | Nonavalent | 90% | 45% |
| S15 | Nonavalent | 90% | 90% |

**Figure S4. Predicted cervical cancer incidence rates in the Netherlands over the period 2020 - 2100 for the different screening scenarios when future vaccination coverage would be 55.2% in girls and 27.6% in boys with a bivalent vaccine. Results are standardized to the WHO standard population.(30)**

**Figure S5. Predicted cervical cancer incidence rates in the Netherlands over the period 2020 - 2100 for the different screening scenarios when future vaccination coverage would be 60% in girls and 30% in boys with a bivalent vaccine. Results are standardized to the WHO standard population.(30)**

**Figure S6. Predicted cervical cancer incidence rates in the Netherlands over the period 2020 - 2100 for the different screening scenarios when future vaccination coverage would be 70% in girls and 35% in boys with a bivalent vaccine. Results are standardized to the WHO standard population.(30)**

**Figure S7. Predicted cervical cancer incidence rates in the Netherlands over the period 2020 - 2100 for the different screening scenarios when future vaccination coverage would be 80% in girls and 40% in boys with a bivalent vaccine. Results are standardized to the WHO standard population.(30)**

**Figure S8. Predicted cervical cancer incidence rates in the Netherlands over the period 2020 - 2100 for the different screening scenarios when future vaccination coverage would be 90% in girls and 45% in boys with a bivalent vaccine. Results are standardized to the WHO standard population.(30)**

**Figure S9. Predicted cervical cancer incidence rates in the Netherlands over the period 2020 - 2100 for the different screening scenarios when future vaccination coverage in boys and girls would be 90% with a bivalent vaccine. Results are standardized to the WHO standard population.(30)**

* Coverage in boys and girls are both assumed to be 90%, whereas in the other scenarios the coverage in boys is assumed to be 50% of that in girls.

**Figure S10. Predicted cervical cancer incidence rates in the Netherlands over the period 2020 - 2100 for the different screening scenarios when future vaccination coverage would be 55.2% in girls and 27.6% in boys with a nonavalent vaccine. Results are standardized to the WHO standard population.(30)**

**Figure S11. Predicted cervical cancer incidence rates in the Netherlands over the period 2020 - 2100 for the different screening scenarios when future vaccination coverage would be 60% in girls and 30% in boys with a nonavalent vaccine. Results are standardized to the WHO standard population.(30)**

**Figure S12. Predicted cervical cancer incidence rates in the Netherlands over the period 2020 - 2100 for the different screening scenarios when future vaccination coverage would be 70% in girls and 35% in boys with a nonavalent vaccine. Results are standardized to the WHO standard population.(30)**

**Figure S13. Predicted cervical cancer incidence rates in the Netherlands over the period 2020 - 2100 for the different screening scenarios when future vaccination coverage would be 80% in girls and 40% in boys with a nonavalent vaccine. Results are standardized to the WHO standard population.(30)**

**Figure S14. Predicted cervical cancer incidence rates in the Netherlands over the period 2020 - 2100 for the different screening scenarios when future vaccination coverage would be 90% in girls and 45% in boys with a nonavalent vaccine. Results are standardized to the WHO standard population.(30)**

**Figure S15. Predicted cervical cancer incidence rates in the Netherlands over the period 2020 - 2100 for the different screening scenarios when future vaccination coverage in boys and girls would be 90% with a nonavalent vaccine. Results are standardized to the WHO standard population.(30)**

* Coverage in boys and girls are both assumed to be 90%, whereas in the other scenarios the coverage in boys is assumed to be 50% of that in girls.

# NNR by elimination year for all vaccination scenarios

Figures S16-S27 below present for each screening scenario the year in which elimination will be reached and the harms-benefits ratio of screening in terms of number of women needed to refer (NNR) to prevent one cervical cancer death over the period 2022-2100 in the Netherlands. The results are presented in separate graphs by vaccination scenario (Table S6) and include the results of the screening scenarios where a more ambitious goal of 90% screening coverage would be reached.

**Table S6. List of vaccination coverages of all figures that present the NNR of each screening strategy by elimination year.**

| **Figure** | **Vaccine type** | **Coverage in girls after 1 January 2022** | **Coverage in boys after 1 January 2022** |
| --- | --- | --- | --- |
| S16 | Bivalent | 55.2% | 27.6% |
| S17 | Bivalent | 60% | 30% |
| S18 | Bivalent | 70% | 35% |
| S19 | Bivalent | 80% | 40% |
| S20 | Bivalent | 90% | 45% |
| S21 | Bivalent | 90% | 90% |
| S22 | Nonavalent | 55.2% | 27.6% |
| S23 | Nonavalent | 60% | 30% |
| S24 | Nonavalent | 70% | 35% |
| S25 | Nonavalent | 80% | 40% |
| S26 | Nonavalent | 90% | 45% |
| S27 | Nonavalent | 90% | 90% |

**Figure S16. Predicted NNR of cervical cancer screening in the Netherlands over the period 2022 - 2100 by the year in which elimination will be reached for that strategy when future vaccination coverage would be 55.2% in girls and 27.6% in boys with a bivalent vaccine. The horizontal line represents the current NNR to prevent one CC death for unvaccinated cohorts.**

CC, Cervical cancer; NNR, number needed to refer to prevent 1 CC death.

**Figure S17. Predicted NNR of cervical cancer screening in the Netherlands over the period 2022 - 2100 by the year in which elimination will be reached for that strategy when future vaccination coverage would be 60% in girls and 30% in boys with a bivalent vaccine. The horizontal line represents the current NNR to prevent one CC death for unvaccinated cohorts.**

CC, Cervical cancer; NNR, number needed to refer to prevent 1 CC death.

**Figure S18. Predicted NNR of cervical cancer screening in the Netherlands over the period 2022 - 2100 by the year in which elimination will be reached for that strategy when future vaccination coverage would be 70% in girls and 35% in boys with a bivalent vaccine. The horizontal line represents the current NNR to prevent one CC death for unvaccinated cohorts.**

CC, Cervical cancer; NNR, number needed to refer to prevent 1 CC death.

**Figure S19. Predicted NNR of cervical cancer screening in the Netherlands over the period 2022 - 2100 by the year in which elimination will be reached for that strategy when future vaccination coverage would be 80% in girls and 40% in boys with a bivalent vaccine. The horizontal line represents the current NNR to prevent one CC death for unvaccinated cohorts.**

CC, Cervical cancer; NNR, number needed to refer to prevent 1 CC death.

**Figure S20. Predicted NNR of cervical cancer screening in the Netherlands over the period 2022 - 2100 by the year in which elimination will be reached for that strategy when future vaccination coverage would be 90% in girls and 45% in boys with a bivalent vaccine. The horizontal line represents the current NNR to prevent one CC death for unvaccinated cohorts.**

CC, Cervical cancer; NNR, number needed to refer to prevent 1 CC death.

**Figure S21. Predicted NNR of cervical cancer screening in the Netherlands over the period 2022 - 2100 by the year in which elimination will be reached for that strategy when future vaccination coverage in boys and girls would be 90% with a bivalent vaccine. The horizontal line represents the current NNR to prevent one CC death for unvaccinated cohorts.**

CC, Cervical cancer; NNR, number needed to refer to prevent 1 CC death.

* Coverage in boys and girls are both assumed to be 90%, whereas in the other scenarios the coverage in boys is assumed to be 50% of that in girls.

**Figure S22. Predicted NNR of cervical cancer screening in the Netherlands over the period 2022 - 2100 by the year in which elimination will be reached for that strategy when future vaccination coverage would be 55.2% in girls and 27.6% in boys with a nonavalent vaccine. The horizontal line represents the current NNR to prevent one CC death for unvaccinated cohorts.**

CC, Cervical cancer; NNR, number needed to refer to prevent 1 CC death.

**Figure S23. Predicted NNR of cervical cancer screening in the Netherlands over the period 2022 - 2100 by the year in which elimination will be reached for that strategy when future vaccination coverage would be 60% in girls and 30% in boys with a nonavalent vaccine. The horizontal line represents the current NNR to prevent one CC death for unvaccinated cohorts.**

CC, Cervical cancer; NNR, number needed to refer to prevent 1 CC death.

**Figure S24. Predicted NNR of cervical cancer screening in the Netherlands over the period 2022 - 2100 by the year in which elimination will be reached for that strategy when future vaccination coverage would be 70% in girls and 35% in boys with a nonavalent vaccine. The horizontal line represents the current NNR to prevent one CC death for unvaccinated cohorts.**

CC, Cervical cancer; NNR, number needed to refer to prevent 1 CC death.

**Figure S25. Predicted NNR of cervical cancer screening in the Netherlands over the period 2022 - 2100 by the year in which elimination will be reached for that strategy when future vaccination coverage would be 80% in girls and 40% in boys with a nonavalent vaccine. The horizontal line represents the current NNR to prevent one CC death for unvaccinated cohorts.**

CC, Cervical cancer; NNR, number needed to refer to prevent 1 CC death.

**Figure S26. Predicted NNR of cervical cancer screening in the Netherlands over the period 2022 - 2100 by the year in which elimination will be reached for that strategy when future vaccination coverage would be 90% in girls and 45% in boys with a nonavalent vaccine. The horizontal line represents the current NNR to prevent one CC death for unvaccinated cohorts.**

CC, Cervical cancer; NNR, number needed to refer to prevent 1 CC death.

**Figure S27. Predicted NNR of cervical cancer screening in the Netherlands over the period 2022 - 2100 by the year in which elimination will be reached for that strategy when future vaccination coverage in boys and girls would be 90% with a nonavalent vaccine. The horizontal line represents the current NNR to prevent one CC death for unvaccinated cohorts.**

CC, Cervical cancer; NNR, number needed to refer to prevent 1 CC death.

* Coverage in boys and girls are both assumed to be 90%, whereas in the other scenarios the coverage in boys is assumed to be 50% of that in girls.

# NNS by elimination year for all vaccination scenarios

Figures S28-S39 below present for each screening scenario the year in which elimination will be reached and the harms-benefits ratio of screening in terms of number of women needed to screen (NNS) to prevent one cervical cancer death over the period 2022-2100 in the Netherlands. The results are presented in separate graphs by vaccination scenario (Table S7) and include the results of the screening scenarios where a more ambitious goal of 90% screening coverage would be reached.

**Table S7. List of vaccination coverages of all figures that present the NNS of each screening strategy by elimination year.**

| **Figure** | **Vaccine type** | **Coverage in girls after 1 January 2022** | **Coverage in boys after 1 January 2022** |
| --- | --- | --- | --- |
| S16 | Bivalent | 55.2% | 27.6% |
| S17 | Bivalent | 60% | 30% |
| S18 | Bivalent | 70% | 35% |
| S19 | Bivalent | 80% | 40% |
| S20 | Bivalent | 90% | 45% |
| S21 | Bivalent | 90% | 90% |
| S22 | Nonavalent | 55.2% | 27.6% |
| S23 | Nonavalent | 60% | 30% |
| S24 | Nonavalent | 70% | 35% |
| S25 | Nonavalent | 80% | 40% |
| S26 | Nonavalent | 90% | 45% |
| S27 | Nonavalent | 90% | 90% |

**Figure S28. Predicted NNS of cervical cancer screening in the Netherlands over the period 2022 - 2100 by the year in which elimination will be reached for that strategy when future vaccination coverage would be 55.2% in girls and 27.6% in boys with a bivalent vaccine. The horizontal line represents the current NNS to prevent one CC death for unvaccinated cohorts.**

CC, Cervical cancer; NNS, number needed to screen to prevent 1 CC death.

**Figure S29. Predicted NNS of cervical cancer screening in the Netherlands over the period 2022 - 2100 by the year in which elimination will be reached for that strategy when future vaccination coverage would be 60% in girls and 30% in boys with a bivalent vaccine. The horizontal line represents the current NNS to prevent one CC death for unvaccinated cohorts.**

CC, Cervical cancer; NNS, number needed to screen to prevent 1 CC death.

**Figure S30. Predicted NNS of cervical cancer screening in the Netherlands over the period 2022 - 2100 by the year in which elimination will be reached for that strategy when future vaccination coverage would be 70% in girls and 35% in boys with a bivalent vaccine. The horizontal line represents the current NNS to prevent one CC death for unvaccinated cohorts.**

CC, Cervical cancer; NNS, number needed to screen to prevent 1 CC death.

**Figure S31. Predicted NNS of cervical cancer screening in the Netherlands over the period 2022 - 2100 by the year in which elimination will be reached for that strategy when future vaccination coverage would be 80% in girls and 40% in boys with a bivalent vaccine. The horizontal line represents the current NNS to prevent one CC death for unvaccinated cohorts.**

CC, Cervical cancer; NNS, number needed to screen to prevent 1 CC death.

**Figure S32. Predicted NNS of cervical cancer screening in the Netherlands over the period 2022 - 2100 by the year in which elimination will be reached for that strategy when future vaccination coverage would be 90% in girls and 45% in boys with a bivalent vaccine. The horizontal line represents the current NNS to prevent one CC death for unvaccinated cohorts.**

CC, Cervical cancer; NNS, number needed to screen to prevent 1 CC death.

**Figure S33. Predicted NNS of cervical cancer screening in the Netherlands over the period 2022 - 2100 by the year in which elimination will be reached for that strategy when future vaccination coverage in boys and girls would be 90% with a bivalent vaccine. The horizontal line represents the current NNS to prevent one CC death for unvaccinated cohorts.**

CC, Cervical cancer; NNS, number needed to screen to prevent 1 CC death.

* Coverage in boys and girls are both assumed to be 90%, whereas in the other scenarios the coverage in boys is assumed to be 50% of that in girls.

**Figure S34. Predicted NNS of cervical cancer screening in the Netherlands over the period 2022 - 2100 by the year in which elimination will be reached for that strategy when future vaccination coverage would be 55.2% in girls and 27.6% in boys with a nonavalent vaccine. The horizontal line represents the current NNS to prevent one CC death for unvaccinated cohorts.**

CC, Cervical cancer; NNS, number needed to screen to prevent 1 CC death.

**Figure S35. Predicted NNS of cervical cancer screening in the Netherlands over the period 2022 - 2100 by the year in which elimination will be reached for that strategy when future vaccination coverage would be 60% in girls and 30% in boys with a nonavalent vaccine. The horizontal line represents the current NNS to prevent one CC death for unvaccinated cohorts.**

CC, Cervical cancer; NNS, number needed to screen to prevent 1 CC death.

**Figure S36. Predicted NNS of cervical cancer screening in the Netherlands over the period 2022 - 2100 by the year in which elimination will be reached for that strategy when future vaccination coverage would be 70% in girls and 35% in boys with a nonavalent vaccine. The horizontal line represents the current NNS to prevent one CC death for unvaccinated cohorts.**

CC, Cervical cancer; NNS, number needed to screen to prevent 1 CC death.

**Figure S37. Predicted NNS of cervical cancer screening in the Netherlands over the period 2022 - 2100 by the year in which elimination will be reached for that strategy when future vaccination coverage would be 80% in girls and 40% in boys with a nonavalent vaccine. The horizontal line represents the current NNS to prevent one CC death for unvaccinated cohorts.**

CC, Cervical cancer; NNS, number needed to screen to prevent 1 CC death.

**Figure S38. Predicted NNS of cervical cancer screening in the Netherlands over the period 2022 - 2100 by the year in which elimination will be reached for that strategy when future vaccination coverage would be 90% in girls and 45% in boys with a nonavalent vaccine. The horizontal line represents the current NNS to prevent one CC death for unvaccinated cohorts.**

CC, Cervical cancer; NNS, number needed to screen to prevent 1 CC death.

**Figure S39. Predicted NNS of cervical cancer screening in the Netherlands over the period 2022 - 2100 by the year in which elimination will be reached for that strategy when future vaccination coverage in boys and girls would be 90% with a nonavalent vaccine. The horizontal line represents the current NNS to prevent one CC death for unvaccinated cohorts.**

CC, Cervical cancer; NNS, number needed to screen to prevent 1 CC death.

* Coverage in boys and girls are both assumed to be 90%, whereas in the other scenarios the coverage in boys is assumed to be 50% of that in girls.

# Assuming random non-attendance

Table S8 presents the optimal screening strategies for the sensitivity analysis where non-attendance is distributed randomly over the population from 2022 onwards, instead if the assumption that 10% of the population never attends screening. The NNR for the unvaccinated population under this assumption is 76.09

**Table S8. Optimal screening strategies that reach elimination first without exceeding the current NNR, by vaccination scenario.**

| **Vaccination scenario** | | **Screening strategy for vaccinated cohorts^b^** | | **Elimination year (years of delay^c^)** | **Cervical cancer deaths per 100.000 person years over 2022-2100** | |
| --- | --- | --- | --- | --- | --- | --- |
| **Coverage^a^** | **Vaccine type** | **Number of lifetime screens** | **Coverage (%)** |  | **Optimal strategy** | **Difference with base case scenario**^d^ |
| 90%^e^ | 9V | 2^f^ | 70 | 2033 (-2)^g^ | 0.98 | 0.27 (-21%) |
| 90% | 9V | 3^f^ | 70 | 2035 (0) | 0.96 | 0.28 (-23%) |
| 80% | 9V | 3^f^ | 70 | 2035 (0) | 0.98 | 0.27 (-21%) |
| 70% | 9V | 3^f^ | 70 | 2035 (0) | 1.01 | 0.23 (-19%) |
| 60% | 9V | 3^f^ | 70 | 2035 (0) | 1.06 | 0.19 (-15%) |
| 55% | 9V | 3^f^ | 70 | 2035 (0) | 1.10 | 0.15 (-12%) |
| 90%^e^ | 2V | 3 | 70 | 2036 (1) | 1.22 | 0.03 (-2%) |
| 90% | 2V | 1^f^ | 70 | 2036 (1) | 1.29 | -0.04 (3%) |
| 80% | 2V | 1^f^ | 70 | 2036 (1) | 1.28 | -0.04 (3%) |
| 70% | 2V | 0^f^ | 70 | 2036 (1) | 1.40 | -0.16 (13%) |
| 60% | 2V | 0^f^ | 61 | 2038 (3) | 1.50 | -0.25 (20%) |
| 55% | 2V | 0^f^ | 61 | 2038 (3) | 1.51 | -0.26 (21%) |

^a^ Vaccination coverage in girls. Coverage in boys is assumed to be 50% of that in girls unless indicated otherwise.

^b^ Unvaccinated cohorts are screened according to the current guidelines.

^c^ Years of delay compared with the most intense screening strategy (five lifetime screens for all women and 70% screening coverage).

^d^ In the base case scenario vaccination coverage is 55% with the bivalent vaccine. Women receive 5 lifetime screening invitations and screening coverage is 61%.

^e^ 90% coverage in both boys and girls.

^f^ Women in vaccinated cohorts that did not receive vaccination are screened to the current guidelines.

^g^ Elimination is reached two years earlier compared to the most intense screening strategy because some cancer incidences are postponed.

9V, Nonavalent vaccine; 2V, Bivalent vaccine; NNR, number needed to refer to prevent one cervical cancer death.

# Assuming waning vaccination

Table S9 presents the optimal screening strategies for the sensitivity analysis where waning vaccination is assumed. Instead of the assumption that vaccination offers lifetime protection, here we assumed waning efficacy of vaccination after on average 30 years. The NNR for the unvaccinated population under this assumption is equal to that of the base case scenario (82.17).

**Table S9. Optimal screening strategies that reach elimination first without exceeding the current NNR, by vaccination scenario under the assumption of waning vaccine efficacy after on average 30 years.**

| **Vaccination scenario** | | **Screening strategy for vaccinated cohorts^b^** | | **Elimination year (years of delay^c^)** | **Cervical cancer deaths per 100.000 person years over 2022-2100** | |
| --- | --- | --- | --- | --- | --- | --- |
| **Coverage^a^** | **Vaccine type** | **Number of lifetime screens** | **Coverage (%)** |  | **Optimal strategy** | **Difference with base case scenario**^d^ |
| 90%^e^ | 9V | 2^f^ | 70 | 2040 (0) | 1.34 | -0.53 (-28%) |
| 90% | 9V | 2^f^ | 70 | 2040 (0) | 1.55 | -0.33 (-17%) |
| 80% | 9V | 1^f^ | 70 | 2040 (0) | 1.70 | -0.17 (-9%) |
| 70% | 9V | 3^f^ | 70 | 2042 (2) | 1.66 | -0.21 (-11%) |
| 60% | 9V | 3^f^ | 70 | 2042 (2) | 1.75 | -0.12 (-6%) |
| 55% | 9V | 3^f^ | 61 | 2042 (2) | 1.86 | -0.01 (-1%) |
| 90%^e^ | 2V | 1^f^ | 70 | 2040 (0) | 1.60 | -0.27 (-15%) |
| 90% | 2V | 3^f^ | 61 | 2042 (2) | 1.67 | -0.20 (-11%) |
| 80% | 2V | 1^f^ | 70 | 2042 (2) | 1.85 | -0.02 (-1%) |
| 70% | 2V | 3 | 70 | 2043 (3) | 1.85 | -0.02 (-1%) |
| 60% | 2V | 3 | 70 | 2044 (3) | 1.95 | +0.08 (4%) |
| 55% | 2V | 2 | 70 | 2044 (3) | 2.20 | +0.33 (18%) |

^a^ Vaccination coverage in girls. Coverage in boys is assumed to be 50% of that in girls unless indicated otherwise.

^b^ Unvaccinated cohorts are screened according to the current guidelines.

^c^ Years of delay compared with the most intense screening strategy (five lifetime screens for all women and 70% screening coverage).

^d^ In the base case scenario vaccination coverage is 55% with the bivalent vaccine. Women receive 5 lifetime screening invitations and screening coverage is 61%.

^e^ 90% coverage in both boys and girls.

^f^ Women in vaccinated cohorts that did not receive vaccination are screened to the current guidelines.

9V, Nonavalent vaccine; 2V, Bivalent vaccine; NNR, number needed to refer to prevent one cervical cancer death.
