# Supplementary material for: Rapid elimination of cervical cancer while maintaining the harms and benefits ratio of cervical cancer screening: a modelling study
Source: BMC Med. 2022 Nov 9;20:433. doi: 10.1186/s12916-022-02631-7 (PMC9645325; doi:10.1186/s12916-022-02631-7)
Supplement: Supplementary file 2 — Additional file 2. HPV-FRAME reporting checklist. [file 12916_2022_2631_MOESM2_ESM.docx]

HPV-FRAME reporting checklist

The checklist below is based on Canfell et al.(34) who created a quality-based framework for reporting on HPV modelling studies assessing epidemiological and economic policy questions.

|  | **Reported?**  **(Y/N)** | **Reported by age? (Y/N)** | **Report by sex (F-only, M-only or both)?** | **Comments** |
| --- | --- | --- | --- | --- |
| **Inputs** |  |  |  |  |
| Target population for intervention | Y | Y | Y | Methods and Table 1. |
| Sexual behaviour | Y | Y | Y | Additional file 1 page 2 and 3 and references to Matthijsse et al.(18) |
| Cohort examined for evaluation/time horizon | Y | Y | F-only | Unvaccinated and vaccinated cohorts between 1912 and 2100 as described in the Methods. |
| Quality of life assumptions | N/A | N/A | N/A | No cost-effectiveness analysis included. |
| Calibration | Y | Y | F-Only | Additional file 1 page 2-3 and reference to Jansen et al.(14) that contains the calibration results of MISCAN in the model description. |
| Validation (where possible) | Y | Y | F-Only | The model validation was provided in the referenced article(14). |
| Costs | N/A | N/A | N/A | No cost-effectiveness analysis included. |
| Vaccine uptake | Y | Y | Y | Methods and Table 1, age (12) in introduction. |
| Vaccine efficacy | Y | N | N | Reported in methods (95% lifelong). The same efficacy is assumed for males and females. |
| Vaccine cross-protection | Y | N/A | N/A | Model assumes protection for the targeted HPV types only as described in the Methods. |
| Duration vaccine protection and waning | Y | N/A | N | Lifelong protection as described in the Methods. This applies to both males and females. |
| Vaccine and delivery costs | N/A | N/A | N/A | No cost-effectiveness analysis included. |
| Pre-vaccination disease burden (including population attributable fractions for HPV) | Y | Y | F-Only | Type-specific HPV prevalence in STDSIM in Additional file 1 page 2-3. Cervical cancer incidence and mortality rates and HPV-type distribution by lesion grade in referenced paper(14). |
| Duration of natural immunity | Y | N | N | Mentioned in Additional file 1 on page 2 that this is described in Matthijse et al.(18) |
| Routine screening behaviour (routine and follow-up and test-of-cure) | Y | N | F-Only | Methods and Table 1. |
| Screening test(s) and colposcopy accuracies | Y | Does not vary with age | F-Only | Described in referenced paper.(14) |
| Abnormal test management (primary and triage) | Y | Y | F-Only | Invitations for primary tests of current screening programme depend on previous test results as described in referenced paper.(14) |
| Diagnostic follow-up of abnormal tests | Y | Does not vary with age | F-Only | Described in referenced paper.(14) |
| Management by disease grade (confirmed disease) | Y | Does not vary with age | F-Only | Described in referenced paper.(14) |
| Sources of information for screening structure and parameterization | Y | Does not vary with age | F-Only | Described in referenced paper.(14) |
| HPV type incidence, clearance and progression rates | Y | Y | F-Only | All clearance and progression probabilities are presented by age in the referenced paper by Kaljouw et al.(35) |
| Herd effect | N | N | N | The effects of herd-immunity are taken into account by using the dynamic STDSIM model, but are not explicitly presented. |
| Association between vaccination and screening uptake | Y | N/A | N/A | No correlation between vaccination status and screening uptake was assumed, as described in the Methods. |
| Fixed – variable costs[∗](https://www.sciencedirect.com/science/article/pii/S2405852119300230?via%3Dihub#tblA10fnlowast) | N/A | N/A | N/A | No cost-effectiveness analysis included. |
| **Outputs** |  |  |  |  |
| Cancer incidence, mortality, life years, QALYs/DALYs (as appropriate) | Y | N | F-Only | Age specific outputs were used to produce the reported age standardized rates, which are the standard to use for determining the year of cervical cancer elimination. Age specific cancer incidence pre-intervention is presented in referenced paper(14). |
| HPV prevalence, pre-intervention | Y | F-Only | F-Only | Additional file 1 page 2-3 and reference to Jansen et al.(14) that contains the calibration results in the model description. |
| CIN2 detected | N | N | N | Not reported explicitly because this is not the focus of this manuscript. The main health outcome measure is the year of cervical cancer elimination. |
| Sensitivity analysis on key inputs | Y | N | F-Only | Sensitivity analyses have been performed assuming random non-attendance and presented on page 28 of Additional file 1. Furthermore a wide variety of attendances have been simulated including 90% screening uptake, which is presented in Additional file 1 Figures S4-S39. |
| Incremental cost-effectiveness ratios and costs saved | N | N | N | No cost-effectiveness analysis included. |
| Absolute reductions in HPV infections, and/or warts, post-vaccination | N | N | N | Not reported explicitly because this is not the focus of this manuscript. The main health outcome measure is the year of cervical cancer elimination. |
| Absolute reductions in CIN2+ post-vaccination | N | N | N | Not reported explicitly because this is not the focus of this manuscript. The main health outcome measure is the year of cervical cancer elimination. |
| Absolute reductions in invasive cancer (cervical and other HPV cancers, as relevant) post-vaccination | Y | N | F-Only | Cervical cancer incidence rates over time are presented in Figure 1. Absolute reductions in cervical cancer mortality rate are presented in Table 2 and for the sensitivity analyses in Additional file 1, Tables S8 and S9. |

**Abbreviations:**

HPV = Human papillomavirus; QALYs = quality adjusted lifeyears; DALYs = disability adjusted lifeyears; CIN = cervical intraepithelial neoplasia.
